# Compensation Mechanism of the Photosynthetic Apparatus in *Arabidopsis thaliana ch1* Mutants

**DOI:** 10.3390/ijms22010221

**Published:** 2020-12-28

**Authors:** Joanna Wójtowicz, Adam K. Jagielski, Agnieszka Mostowska, Katarzyna B. Gieczewska

**Affiliations:** 1 Department of Plant Anatomy and Cytology, Institute of Experimental Plant Biology and Biotechnology, Faculty of Biology, University of Warsaw, I. Miecznikowa 1, 02-096 Warsaw, Poland; j.wojtowicz@biol.uw.edu.pl (J.W.); mostowag@biol.uw.edu.pl (A.M.); 2 Department of Metabolic Regulation, Institute of Biochemistry, Faculty of Biology, University of Warsaw, I. Miecznikowa 1, 02-096 Warsaw, Poland; xleigaj@biol.uw.edu.pl

**Keywords:** *ch1* mutants, *chlorina*, chlorophyll *b* deficiency, chlorophyllide *a* oxygenase, light-harvesting complex, low-light stress

## Abstract

The origin of chlorophyll *b* deficiency is a mutation (*ch1*) in chlorophyllide *a* oxygenase (CAO), the enzyme responsible for Chl *b* synthesis. Regulation of Chl *b* synthesis is essential for understanding the mechanism of plant acclimation to various conditions. Therefore, the main aim of this study was to find the strategy in plants for compensation of low chlorophyll content by characterizing and comparing the performance and spectral properties of the photosynthetic apparatus related to the lipid and protein composition in four selected Arabidopsis *ch1* mutants and two Arabidopsis ecotypes. Mutation in different loci of the CAO gene, *viz*., NW41, *ch1.1*, *ch1.2* and *ch1.3*, manifested itself in a distinct *chlorina* phenotype, pigment and photosynthetic protein composition. Changes in the CAO mRNA levels and chlorophyllide *a* (Chlide *a*) content in ecotypes and *ch1* mutants indicated their significant role in the adjustment mechanism of the photosynthetic apparatus to low-light conditions. Exposure of mutants with a lower chlorophyll *b* content to short-term (1LL) and long-term low-light stress (10LL) enabled showing a shift in the structure of the PSI and PSII complexes via spectral analysis and the thylakoid composition studies. We demonstrated that both ecotypes, Col-1 and Ler-0, reacted to high-light (HL) conditions in a way remarkably resembling the response of *ch1* mutants to normal (NL) conditions. We also presented possible ways of regulating the conversion of chlorophyll *a* to *b* depending on the type of light stress conditions.

## 1. Introduction

Plants require light to live, notably to conduct photosynthesis. This process takes place in chloroplasts and converts light energy into chemical energy, which fuels multiple metabolic processes and sustains plant growth. Chloroplasts’ internal membranes, called thylakoids, provide a platform for the light reactions during photosynthesis. The grana and stroma thylakoids contain distinct photosynthetic complexes, which differ not only in abundance but also in their distribution within the photosynthetic membranes. In stacked membranes, the major chlorophyll *a/b* light-harvesting complexes (LHCII) and minor light-harvesting complexes (Lhcb4, Lhcb5, Lhcb6) form the LHCII-PSII supercomplex with the photosystem II dimer (PSII). Moreover, the LHCII-PSII and mobile LHCII trimers build up a less stable macrodomain structure [1]. In intact grana, these complexes have been shown to form densely packed aggregates. In unstacked lamellae, the photosystem I (PSI), composed of twelve subunits and associated with external antenna (Lhca1-4), constitutes the LHCI-PSI supercomplexes [2,3].

Most of the light energy is captured by chlorophyll and carotenoid pigments in chloroplast thylakoids. Chlorophyll *a* (Chl *a*) and chlorophyll *b* (Chl *b*) are differently distributed in various chlorophyll–protein complexes (CP). Chl *a* is present both in photosynthetic reaction centers and the light-harvesting antennae. Chl *b* occurs mainly in the peripheral antenna complexes, while its level is deficient in the PSII core antenna complexes such as CP43, CP47 and P680 Chl complex [4]. The chemical structures of the two primary chlorophyll pigments differ only in one position of the tetrapyrrole ring: Chl *a* has a methyl group in the C7 position, while Chl *b* has a formyl group in the same position [5]. Furthermore, due to different absorption spectra in blue and red regions, plants that exhibit a combination of both Chl *a* and *b* can absorb light of a broader spectrum range [6]. As many reports state, chlorophyll biosynthesis occurs in all vascular plants and green algae [7,8], in which newly synthesized chlorophyll *a* is a substrate for chlorophyll *b* synthesis. This two-step reaction, via the 7-hydroxymethyl chlorophyll *a*, is catalyzed by an enzyme called the chlorophyllide *a* oxygenase (CAO), which is a Rieske–mononuclear iron oxygenase. It was stated that the CAO activity is mostly post-translationally regulated at the protein stability level [9]. Recently published works show that when the CAO gene is overexpressed in tobacco, only minor effects on the chlorophyll *a* to *b* ratios were recorded [10,11]. Similar observations were made when the full-length CAO cDNA was overexpressed in Arabidopsis [9,12]. The CAO protein was reported to localize in the inner envelope and the thylakoid membranes of barley and *A. thaliana* chloroplasts. The last 15 years of research reveal that the CAO protein also regulates the import and stabilization of light-harvesting proteins (LHCs) in thylakoids [13]. Furthermore, the accumulation of LHC proteins in higher plants is strictly determined by chlorophyll *b* biosynthesis. It has been suggested that chlorophyll *b* stabilizes LHC, and therefore LHC contributes to the assembly of grana lamellae [14].

The role of LHC proteins as major components of the thylakoid membranes is fundamental; as light-harvesting Chl *a*/*b* complexes, they bind more than 40% of the total chlorophyll, collect energy and transfer it to PSI and PSII [15]. Moreover, antenna complexes show controlled changes adapting to various growth conditions, enabling optimal utilization of the available light energy and protecting the photosynthetic apparatus from damage. Studies based on electron microscopy reveal that the grana and stroma lamellae are highly malleable to low-light (LL) and high-light (HL) exposure [16]. It was established that the chlorophyll *a* to *b* ratio is lower when plants are grown under LL conditions [17]. In these specific circumstances, chlorophyll *b* synthesis is activated, and as a result, there is an increase in the LHC protein level and antenna size [18]. On the contrary, HL conditions force a decrease in the LHC amount in order to reduce the antenna size and secure the photosynthetic apparatus against high light stress [19]. Studies carried out on CAO-overexpressing plants further supported the hypothesis that chlorophyll *b* biosynthesis regulates LHC levels [20]. Notably, the stronger enzymatic activity of the CAO protein increases the chlorophyll *b* levels, which results in a higher accumulation of major LHCs [18]. Although the effect of CAO overexpression on the LHC levels in normal growth conditions was reported to be small, its impact was more evident under HL conditions where the LHC levels were significantly reduced in the wild type (wt) [4]. Comprehensively, these findings demonstrate that there is a close relation between the CAO enzymatic activity, chlorophyll *b* levels and the distribution of major LHCs in thylakoid membranes. The *chlorina* mutants, with lowered chlorophyll *b* levels, seem to be an appropriate choice to study this relation and the modifications that occur in the thylakoid membranes during stress. *Chlorina* plants have been reported in Arabidopsis [21], barley [22] and rice [23,24]. Chlorophyll *b* deficiency is caused by a mutation (*ch1)* in chlorophyllide *a* oxygenase (CAO), the enzyme responsible for Chl *b* synthesis. It is known that the regulation of Chl *b* synthesis is important for understanding the mechanism of plant acclimation to various conditions. To verify this hypothesis, we decided to identify the possible compensation mechanism(s) of the photosynthetic apparatus during low chlorophyll *b* content. Therefore, we characterized and compared the performance and spectral properties of the photosynthetic apparatus together with the lipid and protein composition of the thylakoid membranes in four selected Arabidopsis *ch1* mutants and two Arabidopsis ecotypes in different light intensities: normal light, which corresponds to control conditions (NL), low-light stress (LL) and high-light stress (HL) conditions. Short- and long-lasting low-light stress (LL) was applied to determine the modifications of thylakoid arrangement among all analyzed plants. Considering that *ch1* mutants exhibit a light-sensitive phenotype, two accessions were also exposed to high-light (HL) stress to examine and establish the possibilities of natural acclimation of the photosynthetic apparatus.

We demonstrated that both ecotypes, Columbia (Col-1) and *Landsberg erecta* (Ler-0), reacted to HL conditions in a way remarkably resembling the response of *ch1* mutants to normal (NL) conditions. We also presented possible ways of regulating the conversion of chlorophyll *a* to *b* during different light stress conditions. These findings may bring us closer to reveal and understand the changes in the photosynthetic apparatus that are responsible for its unique compensation mechanism in the optimal and various light stress conditions.

## 2. Results

### 2.1. Pale Green Phenotypes

Our study was performed on four selected Arabidopsis *ch1* mutants and two Arabidopsis ecotypes. Three of them are allelic *ch1* mutants based on the Col-1 ecotype with mutations in different loci: *ch1.1, ch1.2, ch1.3* (NASC), and one is a *ch1* mutant based on the Ler-0 ecotype with a mutation in the *ch1.1* locus, called NW41.

**Figure 1:**
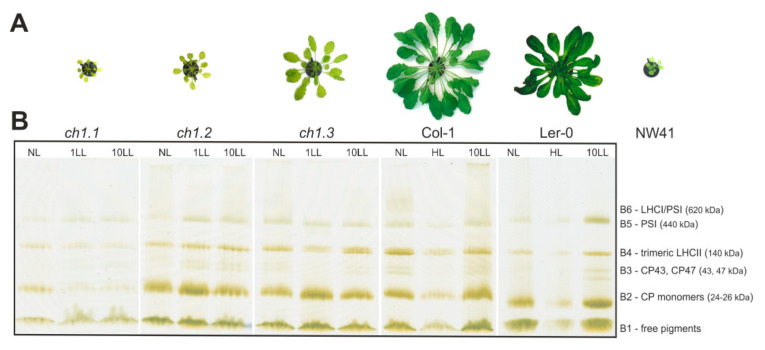
Characterization of Arabidopsis plant phenotypes and analysis of the composition and arrangement of chlorophyll–protein (CP) complexes: (**A**)—morphological phenotypes of 8-week-old mutants *ch1.1*, *ch1.2*, *ch1.3* and NW41 and Arabidopsis ecotypes: Ler-0, Col-1, in normal light (NL) conditions; (**B**)—native PAGE separation of CP complexes from thylakoids of plants grown in NL, low-light (LL) and high-light (HL) conditions; 1LL, 10LL—after 1st and 10th day of treatment; the picture shows true colors; a total of 8.3 µg of total chlorophyll was loaded into each well; interpretation of bands indicated in the figure. NW41 analysis was impossible due to a lack of material with acceptable quality.

The first differences between the *ch1* mutants and selected ecotypes were visible to the naked eye during their growth in normal light conditions: *ch1.1*, *ch1.2* and *ch1.3* displayed a pale green phenotype, differed in the size of the rosette and delayed growth (Figure 1A). The Chl *a*/*b* ratios for three *ch1* mutants in NL conditions indicated that they accumulated reduced amounts of chlorophyll *b* at different levels (Table 1). Meanwhile, *ch1.1* and *ch1.2* mutants did not accumulate Chl *b*; however, *ch1.3* contained a lower level of Chl *b*, compared to the corresponding ecotype in NL. Furthermore, the maximum quantum yield of PSII (F_v_/F_m_) reduced in the *ch1* mutants (Table 1), implying an altered function of the photosynthetic apparatus, which is consistent with previous reports [21,25,26].

To focus and compare the changes appearing in the photosynthetic apparatus of *ch1* mutants with respect to two ecotype lines (Col-1 and Ler-0), different light intensities were applied (normal light, NL—110 μE, representing control conditions; low-light stress, LL—40 μE; and high-light, HL—500 μE). After four weeks of normal growth conditions, *ch1* mutants were switched to LL for ten days, whereas Col-1 and Ler-0 were switched to LL and HL for 10 and 6 days, respectively.

### 2.2. Organization and Composition of the Photosynthetic Apparatus and Analysis of PSI and PSII Protein Levels in Thylakoid Membranes

Several complementary electrophoretic and spectroscopic methods were used for the analysis of the organization of CP complexes (Figure 1B, Figure S1). Mild-denaturing “green” gel electrophoresis enabled a gentle release and separation of the isolated thylakoid membranes of the analyzed plants, revealing six green bands assigned to the chlorophyll–protein complexes (Figure 1B: B1–B6). The most significant changes in the distribution of the bands were visible in all *ch1* mutants in the regions corresponding to the PSII core antenna complex (B3), trimeric forms of LHCII (B4) and the CP monomers (B2) after 1 and 10 days of LL treatment. However, the tendency of these changes depended on the accession plant. Intensities of the B2, B3 and B4 bands increased in 10 LL, probably due to a reduced amount of PSII complexes combined with an increased amount of LHC antenna proteins. An opposite effect was observed in HL conditions, which was consistent with previous reports [16].

**Figure 2:**
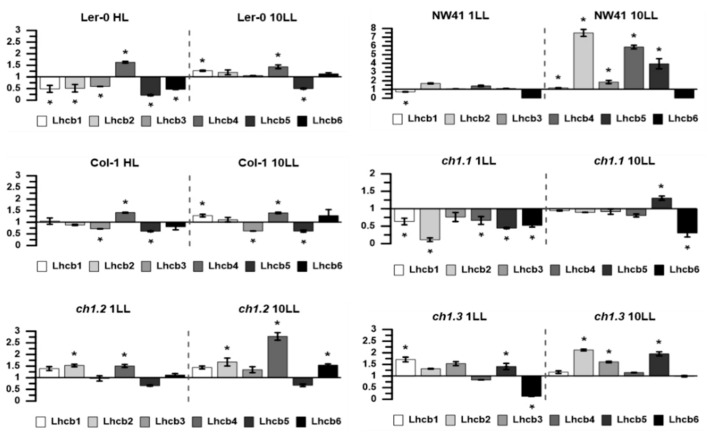
Relative optical density of Lhcb1-6 proteins isolated from NW41, *ch1.1, ch1.2*, *ch1.3*, Ler-0 and Col-1 Arabidopsis plants in LL (low-light) and HL (high-light) conditions; in reference to NL (normal light) conditions; 1LL, 10LL—after 1st and 10th day of treatment. The data were obtained from immunoblot analysis of SDS-PAGE gels with 2 µg of total chlorophyll per well. Presented data are mean values ± SD from 3 independent experiments; pairs of results marked with an asterisk differ significantly at *p* = 0.05 (one-way ANOVA with the post hoc Tukey test).

Precise analysis of the protein composition was conducted based on data obtained from immunodetection. Almost all of the Lhcb protein levels in NL were decreased in the *ch1* mutants compared to Col-1 and Ler-0, except for the Lhcb5 protein detected in the case of *ch1.1*, *ch1.2* and *ch1.3* (Figure S1), which was also reported earlier [7,21,27]. In contrast, the protein levels associated with the PSI antenna were slightly decreased or not markedly changed. Furthermore, LL conditions caused variations in the protein amounts of *ch1* mutants, which confirmed the mild-denaturing electrophoresis results. These variations were visible, especially after the first day of treatment (Figure 1B, Figure 2). In *ch1.2* and *ch1.3* plants, the levels of PSII antenna proteins increased in LL conditions, while *ch1.1* exhibited an opposite effect, and NW41 had almost no effect (Figure 2). The NW41, *ch1.2* and *ch1.3* mutants showed an increase in the Lhcb protein level after long-lasting stress conditions (Figure S1B, Figure 2). Additionally, after 10 days of LL treatment, the amount of PSII core proteins (D1, CP43) decreased in *ch1* mutants except for NW41 (Figure S1B).

**Figure 3:**
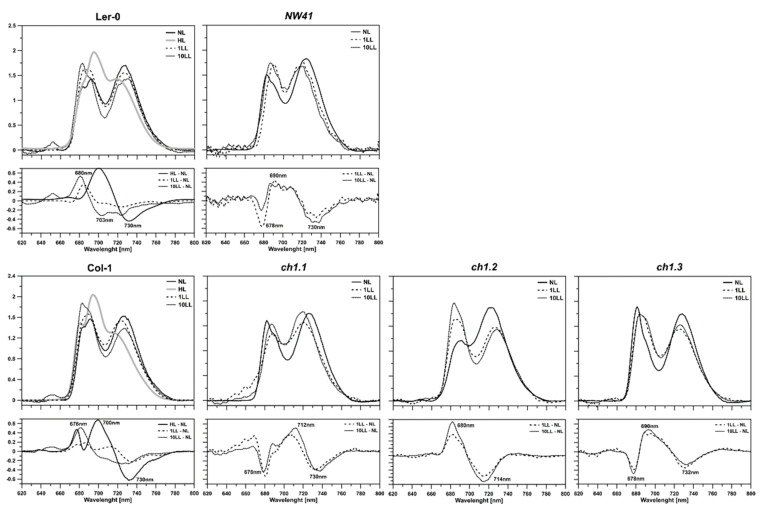
Spectroscopic analysis of thylakoids isolated from NW41, *ch1.1*, *ch1.2*, *ch1.3*, Ler-0 and Col-1 Arabidopsis plants in NL (normal light), LL (low-light) and HL (high-light) conditions; 1LL, 10LL—after 1st and 10th day of treatment. Fluorescence emission spectra at 77K, excited at 470 nm, Chl concentration of 10 μg/mL in 20 mM HEPES-NaOH buffer (pH 7.5) containing 15 mM NaCl, 4 mM MgCl_2_ and 80% (*v*/*v*) glycerol. The spectra were normalized to the area of 100 under the spectrum. The presented spectra are representative of three separate experiments. Fluorescence emission at 685 and 695 nm corresponds to the PSII core and inner antenna, at 730 and 695 nm to the PSI core and inner antenna, at 681 to the LHCII trimers (outer PSII antennae) and at 700 nm to LHCII macroaggregates.

The 77K fluorescence emission spectra pointed out a significantly lower proportion of PSII and LHCII in all *ch1* mutants in NL conditions, compared to the reference plants (Figure 1B, Figure 2, Figure 3, Figure S1). Bands corresponding to LHCII trimers and major antenna proteins: CP43 and CP47, were reduced (Figure 1B), which was consistent with decreased levels of Lhcb1-4 proteins and with the CP43 level obtained by immunodetection (Figure S1B). Interestingly, the 77K fluorescence spectra showed how much the PSI/PSII ratio varied between the mutants in NL (Figure 3). Still, reduced content of LHCII and CP43 (Figure 3, NL; a negative band with the minimum at 682 nm) and increased content of PSI/LHCI were observed (Figure 3, NL; an upbeat band with a maximum at 735 nm), confirming earlier findings [18].

All three *ch1* mutants having mutations in different loci exhibited their specific blueprint for coping with LL conditions.

### 2.3. Gene Expression

**Figure 4:**
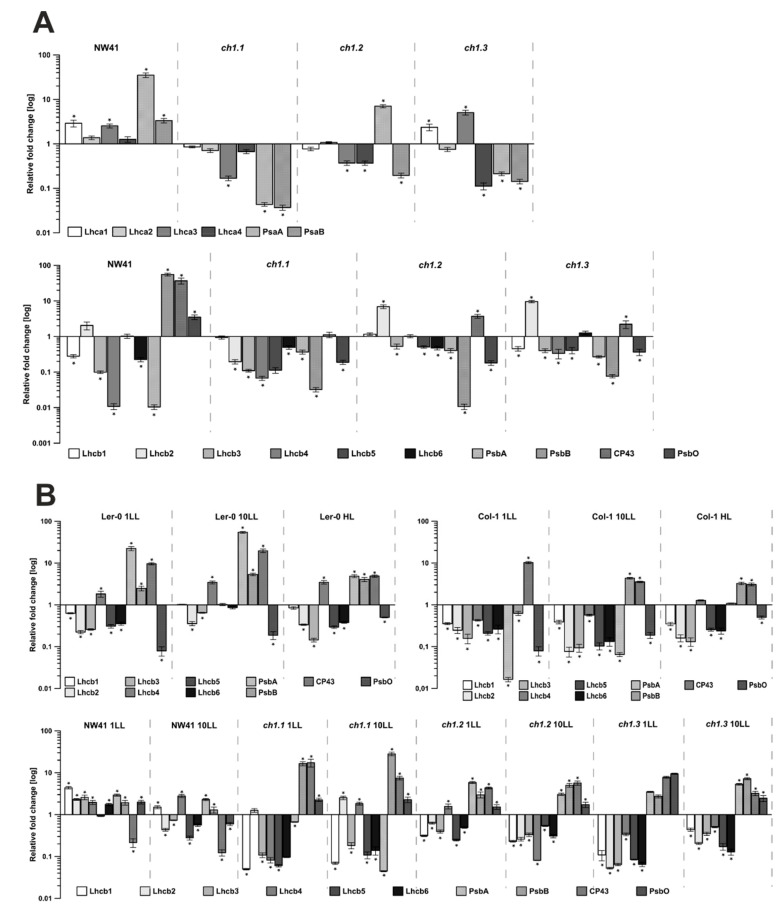
Relative mRNA levels of genes corresponding to antenna and core proteins of PSI and PSII: (**A**)—in reference to the corresponding ecotype in NL (normal light) conditions; (**B**)—in reference to normal growth conditions (NL) of every analyzed Arabidopsis plant in different stress conditions (1LL, 10LL, HL) for PSII genes. LL—low-light conditions (1, 10—after 1st and 10th day of treatment), HL—high-light conditions. The data are mean values ± SD from 3–4 independent experiments; results marked with an asterisk differ significantly at *p* = 0.05 from the corresponding ecotype/plant in NL conditions.

**Figure 5:**
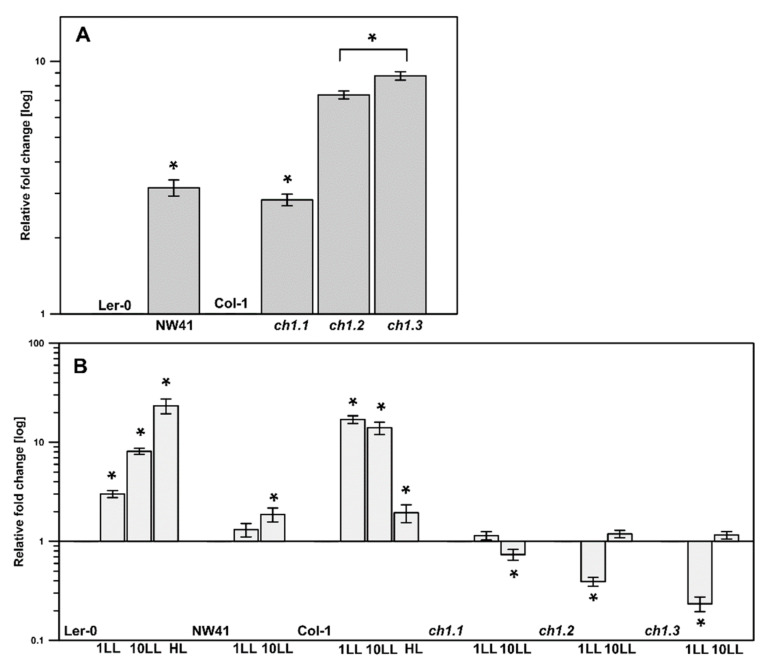
Relative mRNA levels of the CAO gene expression: (**A**)—in reference to the corresponding ecotype in NL (normal light) conditions, (**B**)—in reference to NL conditions for each plant; NL—normal light, LL—low-light conditions (1, 10—after 1st and 10th day of treatment), HL—high-light conditions. The data are mean values ± SD from 3 independent experiments; results marked with an asterisk differ significantly at *p* = 0.05 from the corresponding ecotype/plant in NL conditions.

Chlorophyll *b* synthesis is required for stable integration of the antenna PSII proteins into the thylakoid membrane [27]. Therefore, the genes corresponding to antenna and core proteins of PSII and PSI (Figure 4) and relative mRNA levels of the CAO gene (Figure 5) were determined to investigate their possible correlation with the obtained chlorophyll levels and immunodetection results. The CAO expression levels were increased in all *ch1* mutants in comparison to their corresponding ecotypes in NL conditions; in the case of *ch1.2* and *ch1.3,* the increase was almost 10-fold (Figure 5A). When different light intensities were applied, the CAO expression increased significantly in Col-1 in LL and Ler-0 under both LL and HL conditions (Figure 5B). The *ch1* mutants exhibited an increase in the expression levels in LL, albeit lower than in the corresponding ecotypes (Figure 5B). However, a decrease in the CAO mRNA level was observed in *ch1.2* and *ch1.3* after 1 day of LL and in the *ch1.1* mutant after 10 days of LL treatment as compared to plants grown in NL conditions (Figure 5B). Previous studies indicated that the CAO gene expression is controlled at the transcriptional level and corresponds to changes in light intensity [18]. The results presented in this paper confirm this statement.

The expression of genes corresponding to the core proteins of PSI and PSII was mostly decreased in the *ch1* mutants in NL compared to both ecotypes, except for NW41 (Figure 4A). On the contrary, in low-light conditions (Figure 4B and Figure S2), an increase in these genes was observed in all mutants, which in the case of *ch1.1*, *ch1.2* and *ch1.3* was consistent with the immunoblot results (Figure S1). Additionally, Ler-0 showed an increase in the expression of core proteins of both complexes in LL and HL (Figure 4B and Figure S2). Col-1 had higher levels of mRNA corresponding to core proteins of PSI at 1LL followed by a slight decrease at 10 LL, while a reduction in the mRNA levels corresponding to the D1 protein of PSII was noted. At HL, the expression value of genes attributed to both PSI and PSII core proteins was increased.

The expression levels of genes corresponding to PSII antenna proteins in NL were decreased in all *ch1* mutants (Figure 4A). In contrast, some of the genes corresponding to PSI were increased in the case of *ch1.3* and especially in NW41 (Figure 4A). Low-light conditions caused a decrease in the expression values correlated with the Lhcb proteins in the *ch1* mutants even further, while most of the mRNA levels of the PSI antenna proteins increased significantly (Figure 4B and Figure S2). Interestingly, Ler-0 also displayed a higher amount of mRNA correlated with the PSI antenna proteins in LL, as in HL conditions. In Col-1, the expression values of the majority of genes corresponding to the PSII antenna proteins were decreased in LL and HL.

### 2.4. Efficiency of the Photosynthetic Apparatus

The main parameters of chlorophyll fluorescence in vivo were measured for all analyzed plants. The maximum quantum yield of PSII (F_v_/F_m_) in NL conditions was above the value of 0.8 in both ecotypes (Table 1), which was equal to the optimal value determined earlier for non-stressed wild-type plants [28]. On the contrary, all of the *ch1* mutants exhibited a lower F_v_/F_m_ value. The F_0_ parameter represents the level of fluorescence when Q_A_ acceptors are entirely oxidized and all PSII reaction centers are open [29]. The increase in this value in NW41 and *ch1.3* mutants (Table 1) in comparison to their corresponding ecotypes resulted in a decrease in both efficiencies of the energy transfer between carotenoids in PSII and in the energy absorption efficiency of PSII, which is caused by the disconnection of LHCII antennas from the PSII core. The maximal fluorescence value (F_m_) was higher in NW41 compared to Ler-0, while in *ch1.1*, *ch1.2* and *ch1.3*, it was lower in comparison to their reference ecotypes (Table 1). The PSI quantum yield (Y(I)) was higher only in the *ch1.1* mutant, while the effective quantum yield of PSII (Y(II)) was higher in *ch1.1* and *ch1.2* as compared to Col-1 in NL conditions. Further measurements of the fluorescence quenching were performed in different light intensities (Figure S4). The photochemical quenching (qP) shows the proportion of light energy absorbed by PSII to the energy used for photosynthesis. For *ch1.1* and *ch1.2* in NL, the qP curves reached their inflexion points at higher qP values compared to the curve recorded for Col-1. A similar situation was observed in the NW41 mutant and its reference, Ler-0. Non-photochemical quenching (qN) monitors the apparent rate constant for the heat loss from PSII [30]. In NL conditions, both the initial increase in qN and the rate of subsequent PSII relaxation in the *ch1.3* mutant only were similar to these parameters in the corresponding wild-type plant. In LL, the parameters measured for all plants differed drastically from the values recorded in NL as early as after the first day of treatment (Figure S4, 1LL). An increase in qN, as well as a decrease in qP and Fv/Fm parameters, was observed as a result of the applied stress factor. In the case of *ch1.2* and *ch1.3*, the potential efficiency of PSII (Fv/Fm) in 10 LL increased compared to 1 LL but still did not reach the value recorded in NL. Furthermore, the qP and qN values after 10 days of LL were close to these observed in NL conditions in the ecotypes (Figure S4, Col-1, Ler), which probably represents an adjustment to the changed light intensity.

### 2.5. Pigment Composition

The carotenoid composition was analyzed in thylakoid membranes isolated from all examined plants and presented as a relative change in the carotenoid composition in stress conditions (Figure S3: 1LL, 10LL, HL) in comparison to the same plant in NL conditions (Figure S3). In Col-1, after 1 day of LL treatment, a substantial increase in neoxanthin and violaxanthin levels was observed, while the levels of lutein and zeaxanthin decreased. In 10LL, the trend was different—an increase in neoxanthin, zeaxanthin and carotene compared to the NL conditions was noted (Figure S3; Col-1). After HL treatment, a decline in all measured carotenoids was observed in both ecotypes (Figure S3: Col-1, Ler-0). The obtained data show a similar change in the pigment composition in both ecotypes after 1 day of LL with an additional, almost 1.5 times higher amount of carotene in the case of Ler-0. However, at 10LL, the level of measured carotenoids decreased (Figure S3: Ler-0). The response of *ch1* mutants to stress conditions revealed four diverse compositions compared to their references. In the *ch1.1* mutant, the pool of all analyzed carotenoids was signally reduced at 1LL and 10LL, in contrast with the *ch1.2* mutant, in which a relevant increase was observed. The most noticeable change was the 13-fold increase in lutein content noted at 10LL (Figure S3). The *ch1.3* mutant showed a decreased amount of all analyzed carotenoids at the 1LL stage. NW41 showed an increase only in the violaxanthin pool at 1LL. However, the violaxanthin pool significantly decreased after 10 days of LL treatment, which was coupled with a visible rise in zeaxanthin content compared to NL conditions.

**Figure 6:**
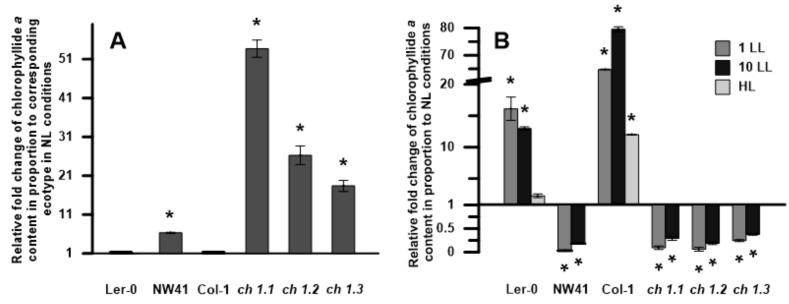
Characterization of chlorophyllide *a* content in thylakoid extracts of analyzed plants: (**A**)—in proportion to the corresponding ecotype in NL conditions; (**B**)—in proportion to normal growth conditions (NL) of every analyzed Arabidopsis plant in different stress conditions (1LL, 10LL, HL) separately. NL—normal light, LL—low-light conditions (1, 10—after 1st and 10th day of treatment), HL—high-light conditions. The data were obtained by HPLC analysis. The data are mean values ± SD from 3 independent experiments; results marked with an asterisk differ significantly at *p* = 0.05 from the corresponding ecotype/plant in NL conditions.

**Figure 7:**
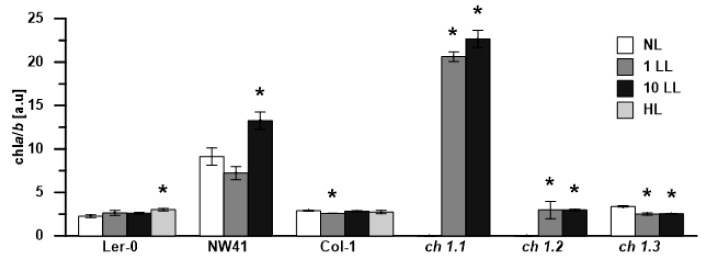
Changes in the Chla/b ratio in thylakoids isolated from all analyzed plants in NL (normal light), LL (low-light) and HL (high-light) conditions. The data were obtained by HPLC analysis. The data are mean values ± SD from 3 independent experiments; results marked with an asterisk differ significantly at *p* = 0.05 from each plant in NL conditions.

The chlorophyllide *a* content (Figure 6A,B) and Chl *a*/*b* ratio (Figure 7) were calculated. Chlide *a* is a polar precursor of chlorophyll *a*, deprived of the phytyl ester group. Figure 6A shows that in NL conditions, the Chlide *a* content was significantly higher in all *ch1* mutants in comparison to their corresponding references. When different light intensities were applied (Figure 6B), the Chlide *a* content shifted. In the case of Col-1 and Ler-0, it was much higher after 1 and 10 days of LL as well as after HL treatment compared to NL conditions. On the contrary, all the *ch1* mutants exhibited a similar decrease in the Chlide *a* content after low-light treatment.

The Chl *a*/*b* ratios in Ler-0 increased slightly in HL (Figure 7, Ler-0 HL), whereas in Col-0, they decreased in LL conditions, which is consistent with earlier reports [26]. Interestingly, in NW41, the plant with a mutation in the same loci as *ch1.1*, the ratio drastically increased during the LL treatment (Figure 7). Meanwhile, in the *ch1.1* and *ch1.2* mutants, chlorophyll *b* was not detected in NL, and the ratio was not calculated until LL conditions were applied (Figure 7). Moreover, the Chl *a*/*b* ratio also increased significantly in the *ch1.2* mutant, reaching the value of 2.97 and 3.0 in 1LL and 10LL, respectively. The *ch1.3* mutant responded to low-light conditions with a decrease in the Chl *a*/*b* ratio, as previously observed in Col-1 after 1LL.

## 3. Discussion

In this study, we examined and characterized the arrangement and distribution, as well as the composition and functionality, of photosynthetic complexes of four Arabidopsis *ch1* mutants (*ch1.1*, *ch1.2*, *ch1.3*, NW41) based on different background lines (Col-1, Ler-0).

### 3.1. Fifty Shades of Pale Green Phenotypes

The characteristics of *chlorina*-type mutants were widely studied due to the importance of chlorophyll *b* as a crucial pigment, connected with numerous physiological processes in plants, e.g., acclimation to light intensity, LHC assembly and degradation or seed maturation [4]. All of the known Chl *b*-deficient mutants of barley, rice and Arabidopsis share characteristic visual features indicating mutations in the CAO gene(s), namely, pale green leaves, delayed growth and smaller plant size (Figure 1A) [23,31,32]. The existing literature concerning Arabidopsis mutants interesting for our studies deals with three *ch1* mutants derived from the Col-1 ecotype with mutations in different loci: *ch1.1*, *ch1.2*, *ch1.3* [5,12,27]. Accordingly, *ch1.1* seemed to attract most of the scientific attention, being a useful literature reference as it, according to reports, contained the least Chl *b* [8,9,18,21,33]. Furthermore, the NW41 mutant examined in this study is an equivalent of *ch1.1* but based on the Ler-0 ecotype, as opposed to the Col-1 ecotype. NW41 is also the oldest [32] and least studied representative of the Arabidopsis *ch1* family. As we showed in this study, its response to light stress conditions does not coincide with the corresponding *ch1.1* mutant (Figure 2 and Figure 3, Figures S1 and S4).

In normal growth conditions, significant differences between all *ch1* mutants were observed. The Chl *a*/*b* ratio could not be detected in pale green *ch1.1* and *ch1.2* but was measured higher in the case of NW41 as compared to both reference plants (Figure 7), which was consistent with previous reports for *ch1* loci [18]. However, the Chl *a*/*b* ratio obtained for *ch1.3* (Table 1) was not consistent with literature data [27], despite the plant’s matching phenotype. Previous studies showed that *ch1.1* and *ch1.3* mutants almost entirely lack Chl *b* [7,21], while *ch1.2* accumulates as much as 20% of Chl *b* as the wild type [34]. Moreover, the increased value of the ratio after LL treatment, observed only in the *ch1* mutants, is worthy of attention (Figure 7). Many studies proved that wt plants in low-light conditions contained a lower amount of PSII complexes and larger PSII antennae [26]; therefore, the Chl *a*/*b* ratio was slightly reduced (Figure 7, Col-1). As mentioned before, Chl *b* is restricted to antenna complexes, whereas Chl *a* occurs both in the photosynthetic reaction centers and the light-harvesting antennae. Furthermore, it was previously reported that the PSI-LHCI supercomplex could be present in *ch1* mutants but in an unstable form and therefore detected as dissociated monomeric forms of PSI and LHCI [21]. Our results contradicted these statements for all analyzed *ch1* mutants (Figure 1B, Figure 2, Figure 4, Figure S1B) as they confirmed the presence of PSI-LHCI components nearly unaffected. The levels of Lhca 1-4 proteins obtained via immunoblot analysis in NL were only slightly lower than in the Col-1 ecotype. Interestingly, Lhcb5 was the only PSII antenna protein detected in significant amounts in almost all analyzed *chlorina*-type plants in NL (Figure S1B). It was known that this particular member of the Lhcb protein group reacts abnormally when the thylakoid pigment composition is disrupted. It was speculated that, even at a low Chl *b* concentration but in the presence of the high amount of the β-β-xanthophylls in the thylakoid membranes, the unfolded apoprotein of Lhcb5 in *ch1* mutants could be stabilized [35]. The studies performed on mutants lacking carotene-derived xanthophylls showed reduced amounts (violaxanthin and zeaxanthin) [36] or absence (neoxanthin) [35] of the Lhcb5 protein. Due to the reported loss of LHCII [37] as well as the absence/reduced values of Lhcb proteins (Figure S1B) in *ch1* plants, a lower amount of neoxanthin, violaxanthin and lutein as compared to their reference plant was expected. However, in our studies, we observed the presence of β-β-xanthophylls, lutein and carotene in thylakoids of all *ch1* mutants in NL conditions (Figure S5).

### 3.2. Same Shortage, Different Statement (Coping with It)

The diversity of results for *ch1* plants presented in this paper suggests a peculiar regulation exhibited by their photosynthetic apparatus in normal light conditions (NL—110 μE). The standard intensity chosen as a reference (NL) in this study did not harm the ecotypes; however, it affected the *ch1* mutants. This light intensity turned out to be harmful to the *ch1.2* mutant probably because of the low pigment and chlorophyll *b* concentration (Table 1, Figure S4). The high amount of zeaxanthin and carotene in NW41, *ch1.1* and *ch1.3* was involved in a protective function of these pigments, according to Havaux et al., 2007. In this regard, the *ch1.3* mutant, despite exhibiting a typical *ch1* phenotype, reacted in a way similar to the Col-1 ecotype (Figure S4). In the heterozygous *ch1.3* mutant, we noticed the appearance of Chl *b* and all the necessary components of the photosynthetic apparatus (Figure 7). However, according to Espineda and coworkers [27] in the homozygous version with a complete null mutation, there should be no chlorophyll *b.*

**Figure 8:**
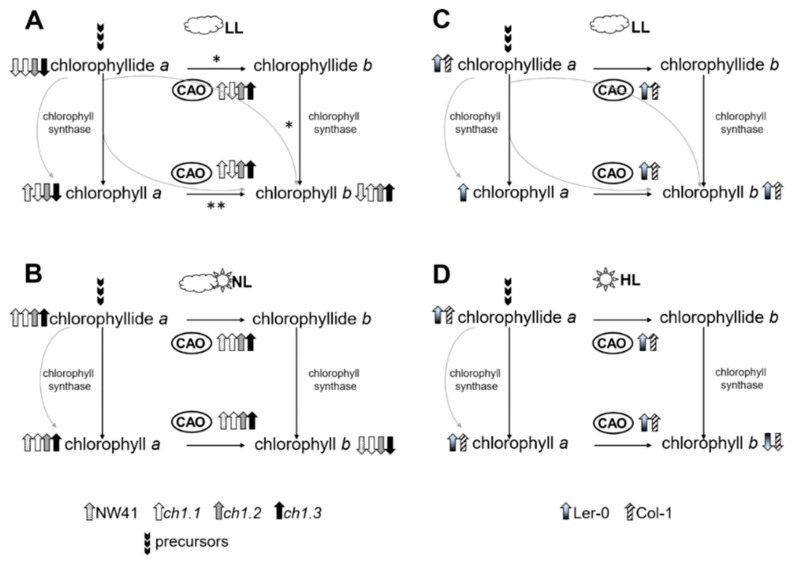
Routes of chlorophyll *a* to *b* conversion. Two possible routes of chlorophyll *a* to *b* conversion were previously described (Tanaka and Tanaka, 2019). In the first route (*), chlorophyll *a* is dephytilated by chlorophyllase and then converted to chlorophyll *b* by chlorophyllide *a* oxygenase (CAO) and chlorophyll synthase. In the second route (**), chlorophyll *a* is directly converted to chlorophyll *b* by CAO activity. Both of the routes were presented separately for all investigated plants in the applied light conditions: normal light (NL), low-light (LL), high-light (HL). (**A**)—for *ch1* mutants: NW41, *ch1.1*, *ch1.2*, *ch1.3*, in LL; (**B**)—for *ch1* mutants: NW41, *ch1.1*, *ch1.2*, *ch1.3*, in NL; (**C**)—control plants: Ler-0 and Col-1, in LL; (**D**)—control plants: Ler-0 and Col-1, in HL. Arrows correspond to the increase/decrease in a specified chlorophyllide/chlorophyll amount and level of CAO mRNA in response to the applied light conditions.

The low-light treatment results differ from the NL response to stress in all analyzed plants. Each of them adopted a unique blueprint for coping with low-light conditions. This study, like the Espineda et al., 1999 paper, revealed several times greater levels of the CAO mRNA in *ch1* mutants in NL than in both ecotypes (Figure 5A) [27]. Earlier results stated that CAO gene expression was strictly regulated by light intensity [20]: up-regulated when plants were transferred from HL to LL and down-regulated when treated conversely [18,38]. Therefore, we anticipated similar changes in the LL conditions. Our findings obtained in the LL conditions for both reference plants (Figure 5B, Col-1, Ler-0) were consistent with the literature data. The CAO expression, presented in Figure 8C, increased after 1LL and 10LL and was accompanied by an increased content of the PSII antenna (Figure 1B; B4, Figure 3) and Lhcb1, Lhcb2 and Lhcb4 proteins (Figure S1). However, both ecotypes did differ in their response to low-light stress, especially in the level of PSI and PSII core gene expression after 1LL and 10 LL; these levels increased in Ler-0 and decreased in Col-1 (Figure 4B). A similar pattern was observed in the 77K spectra (Figure 3)—an increase in the contribution of wavelengths corresponding to the antenna region to the total fluorescence energy after 1LL, followed by an adjustment after 10 LL, resulting in intact cores and antennae (Figure 1B, Ler-0 10LL, B5, B2).

Therefore, the common features to both ecotypes under low-light conditions are as follows: an increase in the level of CAO expression (although in Col-1 it is much higher), the functional connectivity of the photosynthetic complexes and the amount of antenna proteins detected and photosynthetic efficiency in plants in vivo. It is known that the antenna size regulation is one of the crucial strategies applied by plants to acclimate to changing light environments [26], as we have observed. Moreover, the initial increase in antenna content in both ecotypes after 1 LL (Figure S3; Ler-0, Col-1, 1LL) was accompanied by a higher level of neoxanthin and violaxanthin. High carotene and Chlide *a* content combined with a lowered Fv/Fm ratio indicated the occurrence of light stress after 1LL in both Ler-0 and Col-1 (Figure S3, 6B and Figure S4). After 10 days of LL, the functional parameters rose (Figure S4; qP, F_v_/F_m_), reaching almost the NL values. However, the pigment level decreased in Ler-0, probably as an adjustment to a newly established PSII-LHCII and PSI-LHCI proportion. In contrast, the pigment level and the content of PSII antenna proteins increased in Col-1 (Figure 1B, Figure 2 and Figure S3), which matched the increase in the PSII antennae content (Figure 1B, Figure 4 and Figure S1).

The adjustment mechanism of the photosynthetic apparatus is complex and multi-level. The observed changes may be related to the level of phosphorylation of antenna proteins, the availability of photosynthetic pigments and chlorophyll (and thus the activity of enzymes in the chlorophyll synthesis pathway) or regulation at the level of transcription. In this case, one could draw a far-reaching conclusion that in this case, we have two components—regulation at the stage of transcription and the availability of photosynthetic pigments.

Long-lasting (ten days) light stress was applied to *chlorina* plants to check their adaptive abilities in the context of their slower development and reduced rosette diameter. Experiments were conducted on plants grown in hydroponic culture with no sucrose supplementation. Both these factors were the reason for minor discrepancies of the obtained results with some literature data [25,39]. Nevertheless, the methods selected for this study confirmed that the level of CAO mRNA is the primary controller of the LHC accumulation and regulation of its content in response to light intensity and duration.

### 3.3. Chlorina on the Crossroads

Taking into account our results, we would like to propose possible ways of the conversion of chlorophyll *a* to *b* and the role of CAO in this process depending on the type of light stress. The duration of stress conditions is summarized and presented in Figure 8. Two possible routes for the chlorophyll *a* to *b* conversion were described earlier [4,12]. In the first route (Figure 8*), chlorophyll *a* is dephytilated by chlorophyllase and then converted to chlorophyll *b* by CAO and chlorophyll synthase. In the second route (Figure 8**), chlorophyll *a* is directly converted to chlorophyll *b* by CAO. While, in NL conditions, the *ch1* mutants behaved in a way consistent with the literature predictions (Figure 8B), after 1LL and the final 10LL step (Figure 8A), the mutants developed an individual adaptive strategy. We hypothesized that in the case of *ch1.2* and *ch1.3,* a two-stage process took place. Our data indicate that in both mutants, the level of core protein expression and the PSII antenna proportion (Figure 4B, Figures S1 and S2) slightly increased in 1LL. Both plants also exhibited a lowered amount of Chlide *a*, but only in *ch1.2* did the Chl *a/b* ratio increase significantly, while it was not detectable in NL. Therefore, it seemed to trigger the stabilization of the LHC proteins already after the 1LL stage. Furthermore, in 10LL in *ch1.2*, the pigment composition strictly associated with the LHCII antenna proteins (meaning neoxanthin, violaxanthin and lutein) increased (Figure S3) due to the increased amount of PSII antenna proteins (Figure 2, Figure S1).

NW41 and *ch1.1,* except sharing a mutation in the same loci, also demonstrated a very similar response to low light intensity (Figure 8A). High Chl *a/b* ratios (Figure 7, 10LL), an increased core protein gene expression (Figure 4B) and the proportion of PSII and PSI core proteins in 1LL and 10LL explicitly pointed to a shift in these mutants towards CP monomers and a decreased antenna system (Figure 2 and Figure 4). Nominal disparities between both NW41 and *ch1.1* plants occurred, as NW41 had an increased pool of two xanthophyll cycle members (violaxanthin at 1LL and zeaxanthin at the 10LL stage) (Figure S3) and an abnormal qP and qN curve pattern (Figure S4), indicating a difficulty in the NW41 photosynthetic apparatus to adjust to LL conditions. Additionally, *ch1.1* showed a smaller pigment amount and a decreased CAO expression at the 10 LL stage, which was evident in the immunoblot antenna protein results and supported the statement that the LHC accumulation and assembly are regulated by the CAO expression and the presence of chlorophyll *b*. In conclusion, we can assume that the individual response of the chosen *ch1* mutants to LL conditions is involved with the site of the mutation in the CAO gene (ch1.1, ch1.2, ch1.3) and consequently with the different detected level of the CAO gene expression (Figure 5) and Chl a/b ratio (Figure 7). The reported changing values of those parameters after 1LL and 10LL between all mutants lead to distinct LHC protein accumulation (Figure 5 and Figure S1) and a different proportion of CP complexes (Figure 3).

The LHC gene expression results were not consistent with the recent findings of Tanaka and coworkers [4,12]. As shown in Figure 4B, after 1LL and 10LL, the level of Lhcb gene expression was mostly decreased in the *ch1* mutants, except for NW41 exhibiting an increased Lhcb mRNA level. The mRNA level of *Lhcb5* decreased, even though the Lhcb5 protein was the only protein present in NL. Interestingly, in *ch1.3*, as opposed to gene expression, its protein content increased (Figure S1: *ch1.3* 1LL,10LL). It is known that the level of the LHC gene transcription does not correspond to the protein levels [21,40]. The results obtained for both ecotypes confirmed that the relative mRNA levels for Lhcb did not match the expected increased amounts of related antenna proteins (Figure S1 and Figure 3B) in LL conditions [41]. The LHC apoproteins are considered to be synthesized excessively, although antennae are stable only if chlorophylls are connected to them [18]. Furthermore, it was proposed that the LHC accumulation is controlled only by the CAO expression [4]. The CAO protein level was not measured in this study because it was hardly detectable via immunological or mass spectrometry measurements due to its low concentration and volatile distribution in chloroplasts [9].

### 3.4. Natural Abilities to Rearrangements of Photosynthetic Apparatus

Despite the differences as mentioned above between both ecotypes in LL, the switch to HL resulted in a similar response from Col-1 and Ler-0 (Figure 8D). The low-temperature fluorescence spectra showed an increased contribution of the LHCII aggregates not attached to PSII (Figure 3; upbeat band in differential spectra with a maximum at 700 nm) and a partial loss of PSI and PSII core and antenna proteins, confirmed by the native PAGE, immunoblot and mRNA expression levels (Figure 1B, Figure 3B,C, Figure S1). These results suggested dissociation of PSII supercomplexes [4,42,43]. Such drastic rearrangements in the CP complexes also affected the entire pigment content, which decreased significantly in both plants. The F_v_/F_m_ parameter was much lower, probably as a result of loss or dissociation of LHCII from the PSII core and the smaller amount of carotenoids [44]. In contrast, the increased qN parameter indicates high-light stress.

Interestingly, the Chlide *a* content and CAO gene expression level increased slightly (Figure 5 and Figure 6) which was accompanied by the disruption of PSII complexes in HL [42,43]. The lower content of chlorophyll *b* in the studied plants can be correlated with the previously reported lower content of chlorophyll *a* [21] and, in this paper, with the functional image of the photosynthetic apparatus which was visualized in low-temperature fluorescence (Figure 3). The component corresponding to PSI (with the core part in *ch1.1*, *ch1.3* and NW41, and external antennas in *ch1.2*) decreased in all mutants after stress. At the same time, the wild-type plants compensate for high light and its accompanying stress of accumulating aggregated forms of LHCII. Considering the obtained data, we can state that Col-1 and Ler-0 react to HL conditions in a way remarkably resembling the response of *ch1* mutants to NL conditions (Figure 8B,D) which confirms their acute sensitivity to high light intensities.

## 4. Materials and Methods

### 4.1. Plant Material, Growth Conditions, Stress Treatment and Photosynthetic Measurements

*Arabidopsis thaliana* plants (Col-1, Ler-0 and *ch1* mutants obtained from NASC) were grown in hydroponic culture using seed holders (Araponics SA, Liege, Belgium) in custom 1.8-L boxes with low-density support. The seeds were placed on seed holders containing 0.65% (*w*/*v*) agar (Sigma Aldrich Inc., Saint Louis, MO, USA) plugs. The boxes were filled with suitably diluted General Hydroponics solution (GH Flora Series, Hawthorne, Vancouver, WA, USA), and the plants grew under short-day conditions (8-h photoperiod, 110 μE, 24 °C) for about 4 weeks. There was no sucrose supplementation. During experiments, different light intensities were applied: normal light (NL)—110 μE, low-light stress (LL)—40 μE, and high-light stress (HL)—500 μE. After 4 weeks of growth in normal conditions, *ch1* mutants were transferred to LL for 10 days, whereas Col-1 and Ler-0 were transferred to LL and HL for 10 and 6 days, respectively. In vivo chlorophyll *a* fluorescence was measured using a PAM-2000 portable chlorophyll fluorometer (Heinz Walz, Effeltrich, Germany) as described previously [28].

### 4.2. Preparation of Thylakoid Membranes

Thylakoid membranes were isolated by homogenization of leaves in a buffered isotonic medium, as described previously [45]. Thylakoid membranes were always freshly prepared before each experiment and were kept on ice and in the dark for subsequent use. The concentration of chlorophyll (Chl) was quantified spectrophotometrically after extraction with 80% acetone (Merck KGaA, Darmstadt, Germany) [46].

### 4.3. Protein Concentration

A BCA protein assay kit for low concentrations (ab207002, Abcam, Cambridge, UK) was used to quantify the protein content. The assay was performed according to the manufacturer’s instructions. A volume of 50 µL from every thylakoid sample was added to the BCA working reagents, giving a final reaction volume of 200 µL. The reactions were incubated for 120 min at 37 °C. Absorbance was read at 562 nm on a NanoDrop 2000/2000c Spectrophotometer (Thermo Fisher Scientific Inc., Waltham, MA, USA). Protein concentrations were calculated using bovine serum albumin (BSA) standards and a four-parameter logistic curve using the NanoDrop BCA PROTEIN software (v.1.6, Thermo Fisher Scientific Inc., Waltham, MA, USA).

### 4.4. Low-Temperature (77 K) Fluorescence Measurements

Low-temperature (77 K) fluorescence emission spectra were recorded using a modified Shimadzu RF-5301PC spectrofluorometer (Shimadzu Corp., Kioto, Japan) in which the excitation and emission beams are led by optical fibers. Isolated thylakoids were diluted to a Chl concentration of 10 μg/mL in 20 mM HEPES-NaOH buffer (pH 7.5) containing 15 mM NaCl, 4 mM MgCl_2_ and 80% (*v*/*v*) glycerol and placed in a metal cuvette and submerged in liquid nitrogen (all reagents from Avantor Performance Materials Poland S.A., Gliwice, Poland). The excitation wavelength was set at 470 nm, excitation and emission slits were set at 5 nm and scans were taken in the range of 600 to 800 nm. Each spectrum was recorded twice, averaged and background-corrected, and the obtained curve was shifted to 0 at points 640 and 780 nm.

### 4.5. Mild-Denaturing Electrophoresis, SDS-PAGE and Immunoblot Analysis

Isolated thylakoid membranes were prepared and separated via mild-denaturing electrophoresis as described previously [47], with slight modification—samples containing 8.3 µg of chlorophyll were loaded into each well of the stacking gel. Standard SDS-PAGE and transfer conditions were used as described previously [48]. Proteins were detected on a PVDF membrane (Merck Millipore, Burlington, MA, USA) by using primary antibodies (all raised in rabbits, from Agrisera AB, Vännäs, Sweden) against core and antenna proteins of PSII and PSI complexes. Subsequently, the ECL Detection System (Bio-Rad Laboratories Inc., Hercules, CA, USA) was used to detect secondary anti-rabbit antibody conjugated to horseradish peroxidase. Primary and secondary antibodies were diluted according to the manufacturers’ protocols: Agrisera and Bio-Rad, respectively. We used listed antibodies: for PSI—Lhca1 (AS01 005), Lhca2 (AS01 006), Lhca4 (AS01 008), PsaA (AS06 172), PsaB (AS10 695); for PSII—Lhcb1 (AS01 004), Lhcb2 (AS01 003), Lhcb3 (AS01 002), Lhcb4 (AS04 045), Lhcb5 (AS01 009), Lhcb6 (AS01 010), D1 (AS10 704), CP43 (AS11 1787), PsbO (AS05 092); and for the ATPase subunit—Atpβ (AS05 085-10).

### 4.6. Extraction of Pigments

Pigments were extracted as described previously [49]. The HPLC analysis of pigments was carried out by the method from Sztatelman et al. BMC Plant Biology (2015) [50]. An amount of 30 µL of methanol pigment extract was loaded with a loop onto a C-18 column (Bionacom Velocity, 5 microns, 4.6 × 250 mm, BIONACOM LTD, Coventry, UK). Pigments were identified by retention time, compared to standards. The chromatogram analysis and peak retention were conducted using MassLynx software (v.4.1, Waters Corp., Milford, MA, USA).

### 4.7. PCR Analysis

Total RNA was extracted as described previously [51], stored at −80 °C and thawed only shortly before the experiment. Optimal reference genes for analyzed plants UBC10 and ACT8 were selected after [52]. Primer sets were taken from Xu and coworkers [53] or designed using Primer-BLAST (NCBI, Bethesda, MD, USA) and checked for specificity by BLAST searching the *A. thaliana* RefSeq RNA database (Table S1).

Expression analysis was conducted by quantitative PCR in a MyGo Pro Real-Time PCR thermocycler (IT-IS INTERNATIONAL LTD., Stokesley, UK), using SensiFAST One-Step MasterMix for SYBR Green No ROX (Bioline, Meridian Bioscience Inc., Cincinnati, OH, USA) with a recommended thermal profile (45 cycles). Following amplification, a melt curve was performed in the 60–95 °C range, with 0.5 °C steps. Relative gene expression in each sample was calculated with MyGo Pro analysis software (v3.3, IT-IS INTERNATIONAL LTD., Stokesley, UK), and scaled to the calibrator sample (wt control). For each one, we used at least three total RNA isolates from one experiment, which came from three different plants and three independent experiments. Triplicates of each isolate were used as a matrix for One-Step reactions. Intra-assay variation was evaluated by calculating SD errors of arithmetic means of sample replicates.

## 5. Conclusions

Summarizing, we state the following:

The selected *chlorina* mutants, even those with a mutation at the same loci, have a different response to low-light stress.

All mutants, as well as ecotypes, show alternative reorganization and modification of the thylakoid membrane components (proteins and pigments alike) in all light conditions.

The *chlorina* plants can rearrange the entire photosynthetic system to the optimal one to adjust it to the low chlorophyll *b* content.

The response of both ecotypes to high-light conditions resembles the reaction of the *ch1* mutants to the normal light conditions.

The CAO expression levels correlate with the change in the PSI and PSII contribution and with the levels of the Lhcb proteins in the analyzed *ch1* mutants observed in NL and under stress conditions.

The main difference between *ch1* mutants was the alteration in the level of CAO expression, which leads to different LHC proteins accumulation.

## Figures and Tables

**Table 1:** Chlorophyll fluorescence analysis of wild-type and *ch1* mutants. NL—normal light conditions, 110 µE. The data are mean values ± SD from 3–4 independent experiments. ND—the amount of chlorophyll *b* was below a reliable level for the method used, hence the absence of the Chl*a*/*b* ratio.

	Ler-0 NL	NW41 NL	Col-1 NL	*ch1.1* NL	*ch1.2* NL	*ch1.3* NL
**Chl*a*/*b***	2.26	9.11 *	2.88	ND *	ND *	3.37 *
	±0.16	±0.55	±0.04	-	-	±0.08
**Fv/Fm**	0.815	0.747 *	0.825	0.76 *	0.734 *	0.776 *
	±0.01	±0.08	±0.01	±0.01	±0.02	±0.02
**F_0_**	0.435	0.595	0.513	0.146 *	0.255 *	0.587
	±0.10	±0.16	±0.08	±0.08	±0.08	±0.09
**Fm**	2.345	2.408	2.86	0.631 *	0.894 *	2.553
	±0.61	±0.48	±0.42	±0.06	±0.17	±0.05
**Y(II)**	0.494	0.300 *	0.435	0.601	0.542	0.256
	±0.01	±0.17	±0.03	±0.09	±0.10	±0.04
**Y(I)**	0.803	0.552 *	0.723	0.767	0.597	0.640
	±0.02	±0.10	±0.03	±0.02	±0.06	±0.06

* *p* = 0.05 (one-way ANOVA with the post hoc Tukey test) from corresponding ecotype.

## Data Availability

The data presented in this study are available on request from the corresponding author. The data are not publicly available due to UW internal regulation.

## Supplementary Materials

Supplementary Materials can be found at https://www.mdpi.com/1422-0067/22/1/221/s1. Table S1. List of primers used in this research, Figure S1 Immunodetection of selected PSI and PSII proteins from isolated thylakoids of examined plants in: NL – normal-light, LL – low-light conditions (1, 10 – after 1st and 10th day of treatment) for *ch1* mutants; NL – normal-light, LL – low-light conditions (10 – after 10th day of treatment), HL – high-light for ecotypes. (A)-immunodetection of PSI antennae and core proteins; (B)-immunodetection of PSII antenna and core proteins, Atpβ (control marker). Figure S2. Relative mRNA levels of genes corresponding to antenna and core proteins of PSI in reference to normal growth conditions (NL) of every analyzed Arabidopsis plant in different stress conditions (1LL, 10LL, HL). The data are mean values ± SD from 3-4 independent experiments, Figure S3. Composition of carotenoids extracted from isolated thylakoids in analyzed mutants and Arabidopsis ecotypes in LL and HL conditions. The data are mean values ± SD from 3 independent experiments; results marked with an asterisk differ significantly at *P* = 0.05 from the corresponding ecotype, Figure S4. The efficiency of photosynthetic light reactions of NW41, *ch1.1*, *ch1.2*, *ch1.3*, Ler-0 and Col-1 Arabidopsis leaves in NL, LL and HL conditions, Figure S5. Composition of carotenoids extracted from isolated thylakoids in analyzed mutants and Arabidopsis ecotypes in NL conditions. The data are mean values ± SD from 3 independent experiments; results marked with an asterisk differ significantly at *p* = 0.05 from the corresponding ecotype.

## Abbreviations

CAO	Chlorophyll *a* oxigenase
Chl	Chlorophyll
Chlide	Chlorophyllide
LHC	Light-harvesting complex
