# Supplementary material for: Compensation Mechanism of the Photosynthetic Apparatus in Arabidopsis thaliana ch1 Mutants
Source: Int J Mol Sci. 2020 Dec 28;22(1):221. doi: 10.3390/ijms22010221 (PMC7794896; doi:10.3390/ijms22010221)
Supplement: Supplementary file 1 [file ijms-22-00221-s001.pdf]

International Journal of
Molecular Sciences

MDPI

Supplementary Material

Supplementary Table S1. List of primers used in this research.

**Name**	**Gene**	**Forward sequence 5′→ 3′**	**Reverse sequence 5′→ 3′**
psbA	ATCG00020	GCGAAAGCGAAAGCCTATGG	GAGGAGCAGCAATGAATGCG
psbB	ATCG00680	TGTTGGATGGTTAGGCCACC	ATCTCACCCAATTGAGCCCG
psaA	ATCG00350	TAGGCTGAGTAGCAGGAGCA	TGTTTCTATTCAGCGGGCGT
psaB	ATCG00340	TGTGAAACGTTACCCTGCCA	GGGACGAGGTGGTACTTGTG
PSBO1	AT5G66570	TAAATGCTCCGACGCTGTCA	GTCTCAGAGCCACCGTCAAT
lhca1	AT3G54890	CTGAACCACATAGTGCAGATGGA	TGGACGAGAGGTGTTTGGTG
lhca2	AT3G61470	GCTGCTTTCACACCCAAGTG	AAGAGTGTTACGGGGAACCTT
lhca3	AT1G61520	CCGTATCAGCCTCTCCAATGT	CAAGTGCTTGTGCTGCCATT
lhca4	AT3G47470	GGTTCATCGGCCAAAACGTC	CATAGCCCATCGTCCGTTGA
CP43	ATCG00280	CCTTTGGGGGAGAAGGATGG	GCCTCCCCAGACCATACAAG
CAO	AT1G44446	TGCTGAGCTTGTAATGGACC	CACCAAACTGCAGTACCAAAAA

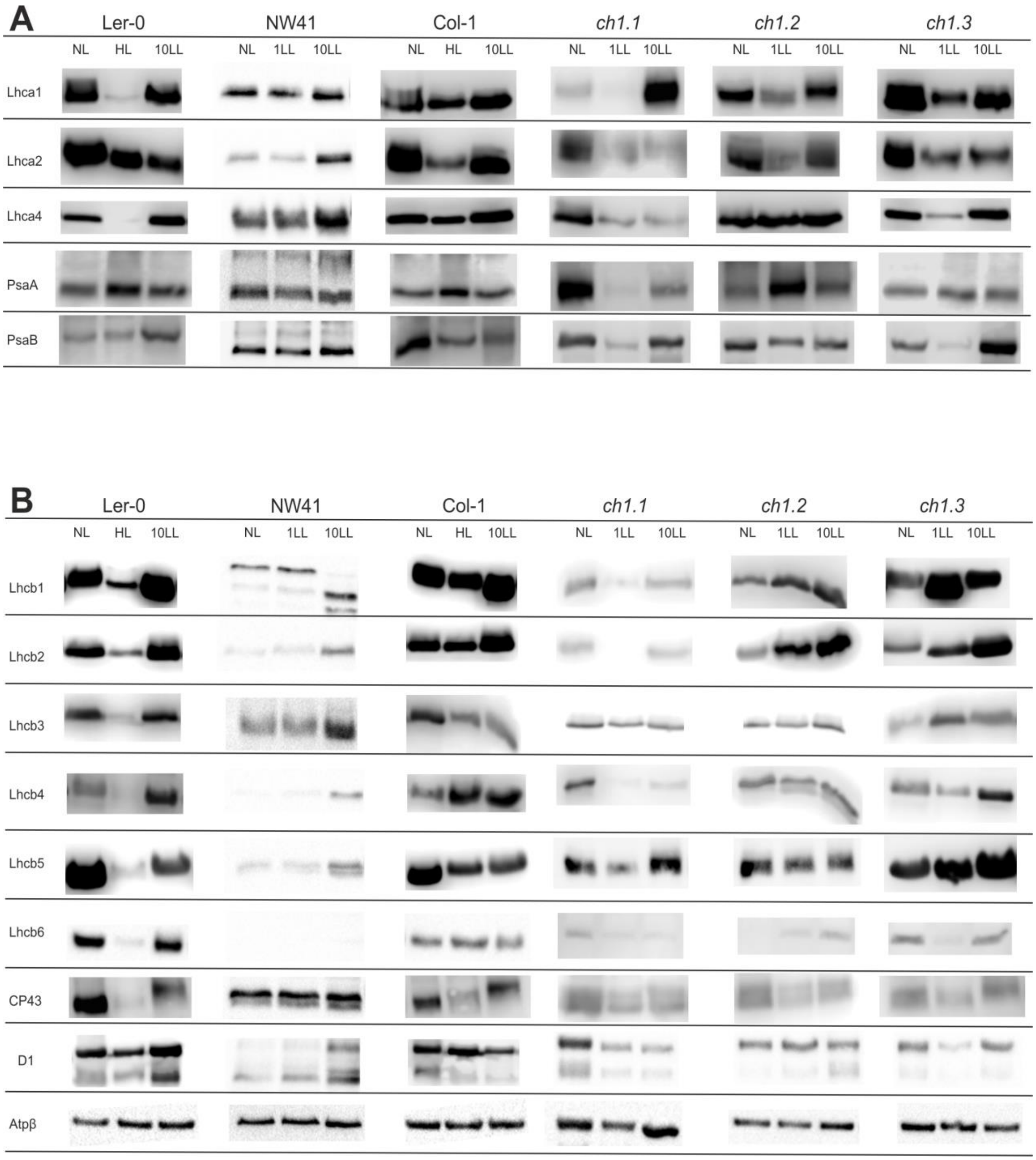

Supplementary Figure S1. Immunodetection of selected PSI and PSII proteins from isolated thylakoids of examined plants in: NL – normal-light, LL – low-light conditions (1, 10 – after 1st and 10th day of treatment) for *ch1* mutants; NL – normal-light, LL – low-light conditions (10 – after 10th day of treatment), HL – high-light for ecotypes. **A**- immunodetection of PSI antennae and core proteins; **B**- immunodetection of PSII antenna and core proteins, Atpβ (control marker).

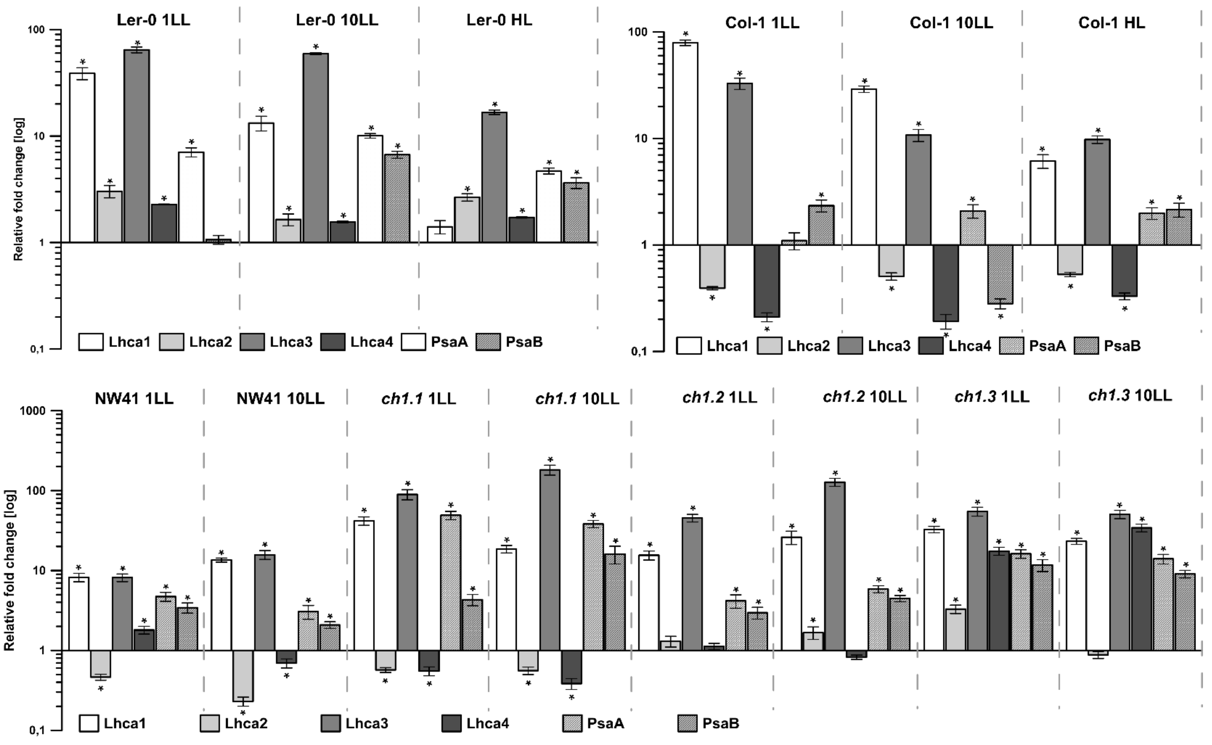

Supplementary Figure S2. Relative mRNA levels of genes corresponding to antenna and core proteins of PSI in reference to normal growth conditions (NL) of every analyzed Arabidopsis plant in different stress conditions (1LL, 10LL, HL). The data are mean values ± SD from 3-4 independent experiments.

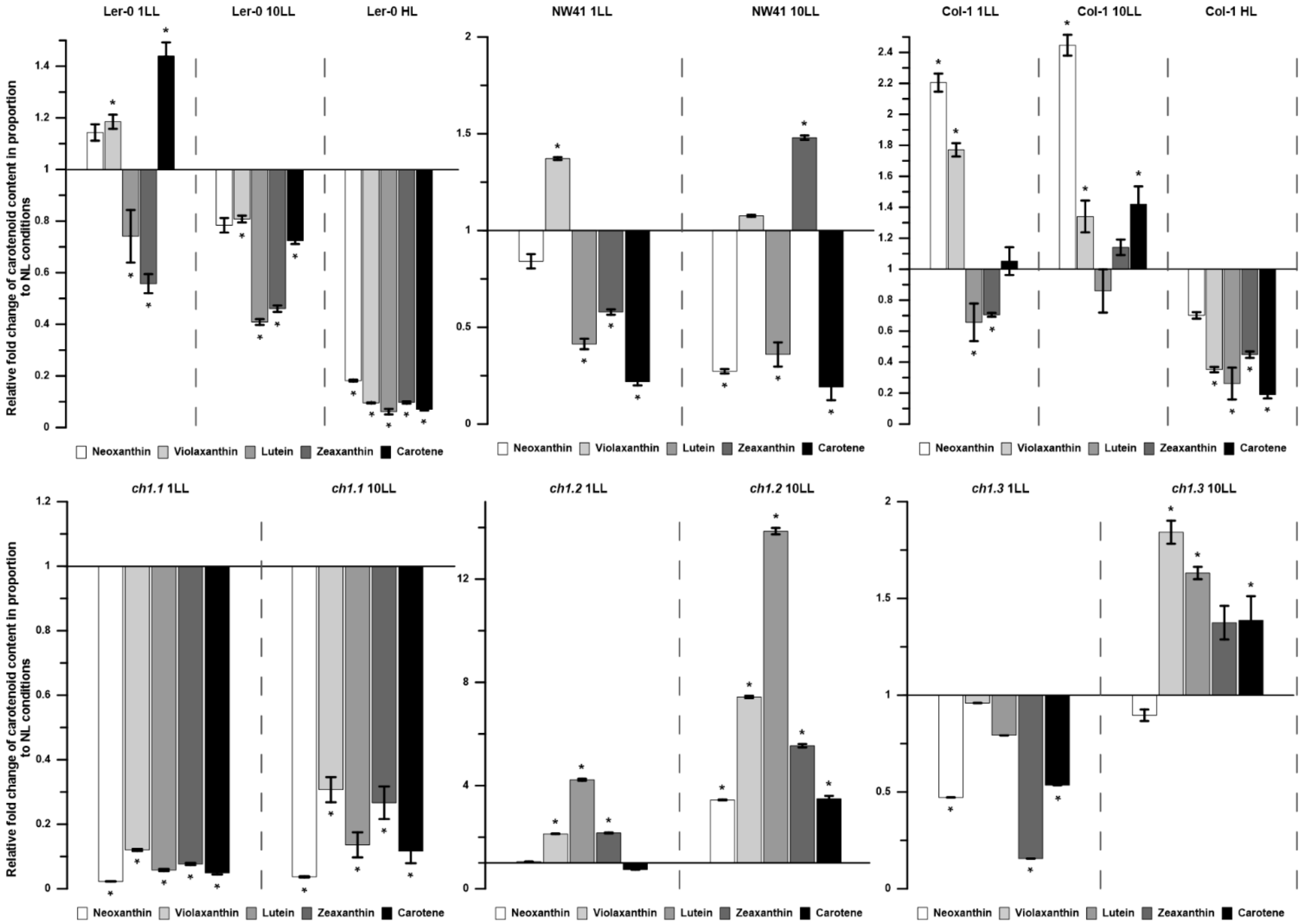

Supplementary Figure S3. Composition of carotenoids extracted from isolated thylakoids in analyzed mutants and *Arabidopsis* ecotypes in LL and HL conditions. The data are mean values ± SD from 3 independent experiments; results marked with an asterisk differ significantly at P = 0.05 from the corresponding ecotype.

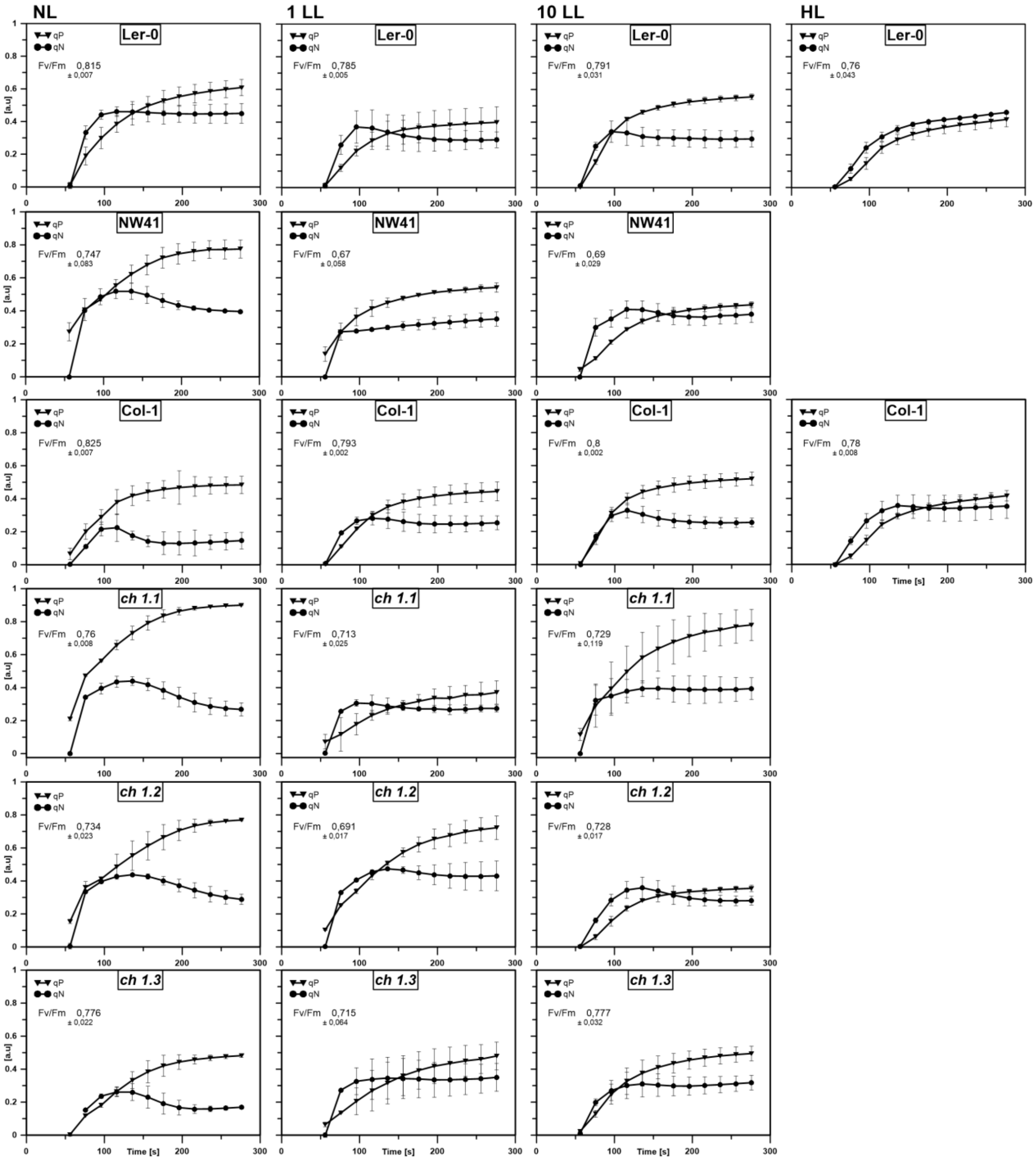

Supplementary Figure S4. The efficiency of photosynthetic light reactions of NW41, *ch1.1*, *ch1.2*, *ch1.3*, Ler-0 and Col-1 *Arabidopsis* leaves in NL, LL and HL conditions.

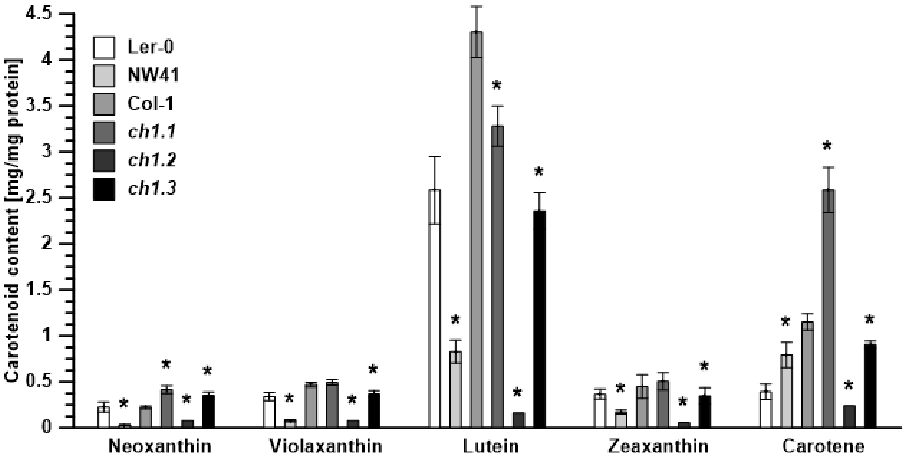

Supplementary Figure S5. Composition of carotenoids extracted from isolated thylakoids in analyzed mutants and Arabidopsis ecotypes in NL conditions. The data are mean values ± SD from 3 independent experiments; results marked with an asterisk differ significantly at P = 0.05 from the corresponding ecotype.
